# Is the emergency MRI protocol for acute pediatric osteoarticular infection a luxury or an absolute priority?

**DOI:** 10.3389/fped.2023.1328870

**Published:** 2023-12-13

**Authors:** Silvia Valisena, Giacomo De Marco, Blaise Cochard, Giorgio Di Laura Frattura, Ludmilla Bazin, Oscar Vazquez, Christina Steiger, Romain Dayer, Dimitri Ceroni

**Affiliations:** ^1^ Pediatric Orthopedics Unit, Pediatric Surgery Service, Geneva University Hospitals, Geneva, Switzerland; ^2^ Division of Orthopedics and Trauma Surgery, Geneva University Hospitals, Geneva, Switzerland

**Keywords:** infection, osteoarticular, pediatric, MRI, diagnostic

## Introduction

Pediatric osteoarticular infections (OAIs) carry significant morbidity if the diagnosis and subsequent treatment are delayed, with potential sepsis, joint damage, growth arrest, and irreversible deformities to the growing skeleton. Over the last 20 years, the advancement of diagnostic technologies, such as nucleic acid amplification technology assays (NAAAs) and the advent of magnetic resonance imaging (MRI), has deepened the knowledge of the etiology and epidemiology of OAIs and, consequently, reshaped the management.

## Towards systematic use of MRI

In the late ‘90ies the rise of MRI technology gradually replaced bone scintigraphy for pediatric OAIs (1). MRI first made its place in the pediatric OAIs workup as an adjunctive exam for the determination of their extent and topography, especially for delayed, complicated or atypic cases (1–4). During the first decade of the new millennium, referral centers have claimed its early use in the workup for both osteomyelitis, septic arthritis, and spondylodiscitis, to facilitate the surgical decision-making process (5–11). During these last years, it has become evident that the quality of care for children with OAIs is significantly impacted by processes that improve the efficiency and accuracy of diagnosis as well as the rapidity and efficacy of treatment. When acute OAIs are suspected in a child, MRI has a primordial role in identifying joint effusion, and locating abscesses, whether osseous (metaphyseal, transphyseal, periosteal, or paraosteal) or in the soft tissues, that can be targeted by the aspiration to achieve definitive diagnosis or be surgically addressed (5, 6, 12–17). Thus, by defining the anatomical and spatial extent of the infection, MRI provides orthopedic surgeons with some of the most precious information to establish the surgical indication, confirm the indication to arthrocenthesis or surgical drainage and finally plan the surgery. MRI for pediatric musculoskeletal infections has a sensitivity of 81%–100% and specificity 67%–94% compared to CT scan (sensitivity of 67%–100% and specificity 50%) (18, 19). MRI allows the visualization of bone marrow oedema, indicating bone abscess formation, and extraosseous spread of the infection. Bone marrow oedema is an early sign of infection, which represents another advantage of MRI. Moreover, with MRI patients do not incur in the radiation exposure risk as for CT scan.

## The difficulty of accessing an emergency MRI

Multiple institutional guidelines have demonstrated that its earlier use remarkably improves the identification of OAIs surgical candidates, thus reducing the rate of sequelae and reoperations (1, 5, 6, 12–16, 20–24). Despite its benefits, the awareness of the resource intensity required by MRI has led to the development of multidisciplinary teams, to expedite the discussion around OAIs management among all the implicated stakeholders, such as pediatricians, orthopedic surgeons, anesthesiologists and radiologists (15, 16, 18, 20, 24–26). When facing OAIs these different specialists might not share the same definition of emergency, with consequent dissent about the diagnostic and therapeutic strategy.

In children aged <4 years old, an MRI usually requires sedation to make the patient lie still during the exam. Moreover, once the diagnosis and the indication for surgical abscess evacuation, debridement, and biopsy are cleared with imaging, the operation must be timely organized, foremostly in the case of MRI under sedation, to benefit from the same anesthesia. To face this challenge, various multidisciplinary teams have allocated dedicated slots in the schedule of the MRI and operating rooms to the care of pediatric OAIs (16, 24). In most of the centers, the efforts were redirected towards obtaining an early MRI, defined as performed <24 h from admission (16, 24, 25). Nevertheless, it is current knowledge that infections, especially septic arthritis, can induce irreversible biological damage to the affected tissues if treatment is delayed by more than 8 h (27). In addition, abrupt onsets with severe systemic involvement in pediatric OAIs are not rare. Seven to 28% of pediatric patients with OAIs may require hospitalization in intensive care unit for accompanying critical illness, in some cases for a septic shock or toxic shock syndrome due to *Staphylococcus aureus* or *Group A beta-hemolytic Streptococcus pyogenes* (28–30).

Nowadays, the criteria for emergency (<6 h) MRI for pediatric OAIs remain an open question (16, 24, 26). In clinically severe OAIs cases, not only the diagnostic work-up must be carried out rapidly, but medical and eventually surgical treatment must be timely begun after the radiologic diagnosis of OAIs has been established (31, 32).

## Indications for emergency MRI

The question about the indications for emergency MRI can be handled in two main ways: by defining which pediatric OAIs are emergencies and what advantage MRI can add in these cases. Real pediatric OAIs emergencies are represented by infections due to pyogenic pathogens, such as *S. aureus*, *Streptococcus pyogenes* or the *Streptococcus pneumoniae* species, that usually present with more severe symptoms and clinical manifestations, and whose clinical course is characterized by a slower clinical response to treatment, with potentially worse outcomes (31, 32). For these reasons, these OAIs require emergency (<6 h) management, justifying invasive diagnostic before intravenous antibiotic treatment, and prompt therapeutic procedures (13, 14). On the opposite, the OAIs due to *K. kingae* are usually characterized by lesser general and local inflammatory reactions and no long-term orthopedic sequelae, making them eligible for antibiotic therapy alone, without surgery (3). The clinical distinction between these two entities can be challenging in some cases but can be corroborated by rapidly available laboratory findings.

## How to discriminate serious OAIs from those due to less aggressive pathogens: laboratory and MRI

When differentiating *K. kingae* from Gram-positive cocci infection, the former is characterized by the following criteria: temperature <38 °C at admission, serum C-reactive protein (CRP) <55 mg/L, white blood cell count <14.000 /mm^3^ and band forms <150 /mm^3^ (31). Similarly, *K. kingae* vs. methicillin-sensitive *S. aureus* OAIs affect patients aged <43 months, with temperature <37.9 °C, CRP <32.5 mg/L, and platelet count >361.500 /mm^3^ at admission (33). Other studies have shown that more than 3 factors among age >4 years, CRP >13.8 mg/L, duration of symptoms >3 days, platelets <314 × 10^3^ cells/ul, neutrophil count >8.6 × 10^3^ cells/ul can predict septic arthritis and represent a good indication for MRI (23, 34). Unfortunately, these criteria were less predictive in other pediatric centers (35). Future research should confirm in multiple settings not only the validity and reliability of these criteria but also their pre-test (*id est*, pre-MRI) predictive value to distinguish among OAIs related to pyogenic vs. non-pyogenic pathogens.

In the case of septic arthritis, preoperative MRI has been demonstrated to allow rapid detection and management of concomitant osteomyelitis, compared to emergency aspiration alone which incurred in 2.8% of reoperation for sequelae (36).

Concerning the distinction between pyogenic vs. non-pyogenic OAIs, MRI has revealed to be a useful tool to discriminate between *K. kingae* and Gram-positive cocci, due to less frequent and less severe bone and soft tissue reactions in the former (13). Despite this, future research should focus on the early recognition of the rare cases of invasive infections due to *K. kingae* (∼1%), characterized by severe complications such as the transphyseal involvement (13, 37–39).

## Towards emergency MRI for pyogenic-related OAIs, and delayed MRI for infections caused by non-aggressive pathogens?

Based on the new epidemiological knowledge of a higher incidence of *K. kingae*-related pediatric OAIs (40–44), it is legitimate to hypothesize that in the future the indication to carry out an emergency (<6 h) or early (<24 h) MRI could no longer appear justified in about 50% of overall cases of OAIs, just by reflecting the incidence of *K. kingae* in the OAIs spectrum (40, 42, 44). In addition, it is now recognized that most *K. kingae* infections are treated with antibiotic therapy alone and do not require surgical interventions (44). On that point, one could even expect that many MRI could be avoided for OAIs whose pre-test (*id est*, pre-MRI) probability to be *K. kingae*-related is high, based on clinical presentation, biological investigations and NAAAs on oropharyngeal specimens (45, 46).

Another field of improvement will also be the development of new MRI protocols to speed up the procedure, avoid sedation and improve the quality of images without the need for contrast (47). The decision-making scheme could be even more efficient when metagenomic next-generation sequencing of microbial cell-free DNA will become an attractive and rapid diagnostic modality for detecting focal OAIs, allowing rapid broad-range pathogen detection by noninvasive sampling such as peripheral blood (the so-called liquid biopsy) (48). It is therefore legitimate to expect the following years to be certainly marked by a revolution in the diagnostic approach and the treatment of OAIs in the pediatric population.
